# The Impact and Successes of a Paediatric Endocrinology Fellowship Program in Africa

**DOI:** 10.1155/2016/1560248

**Published:** 2016-01-20

**Authors:** Gordon Otieno Odundo, Thomas Ngwiri, Olivia Otuoma, Paul Laigong, Renson Mukhwana, Mary Slessor Limbe, Nadia Musimbi Chanzu

**Affiliations:** ^1^Gertrude's Children's Hospital, P.O. Box 42325, Nairobi 00100, Kenya; ^2^Gertrude's Hospital Foundation, Gertrude's Children's Hospital, P.O. Box 42325, Nairobi 00100, Kenya; ^3^Department of Paediatrics and Child Health, University of Nairobi, P.O. Box 460, Nairobi 00202, Kenya; ^4^Department of Paediatrics and Child Health, Aga Khan University, P.O. Box 30270, Nairobi 00100, Kenya; ^5^Institute of Child Health and Research, Gertrude's Children's Hospital, P.O. Box 42325, Nairobi 00100, Kenya

## Abstract

*Background.* The prevalence and distribution of endocrine disorders in children in Africa are not well known because most cases are often undiagnosed or diagnosed too late. The awareness of this led to the launch of the Paediatric Endocrinology Training Center for Africa (PETCA) designed to improve quality and access to health care by training paediatricians from Africa in paediatric endocrinology.* Methods.* The fellowship is undertaken over an 18-month period: six months of clinical and theoretical training in Kenya, nine months of project research at the fellow's home country, and three months of consolidation in Kenya. Upon completion, certified paediatricians are expected to set up centers of excellence.* Results.* There have been two phases, phase I from January 2008 to October 2012 and phase II from January 2012 to April 2015. Fifty-four fellows from 12 African countries have been certified, 34 (phase I) and 20 (phase II). Over 1,000 patients with wide ranging diabetes and endocrine disorders have been diagnosed and treated and are being followed up at the centers of excellence.* Conclusion.* The successes of the PETCA initiative demonstrate the impact a capacity building and knowledge transfer model can have on people in resource-poor settings using limited resources.

## 1. Introduction

Paediatric endocrinology relates to the diagnosis, management, and treatment of diseases of the endocrine system in children and adolescents [1]. These include diabetes, disorders of growth, thyroid function, adrenals and sexual development, obesity, and related complications as well as endocrine cancers [1–7]. The prevalence rates of disorders linked to paediatric endocrinology are underappreciated especially in low and middle-income countries (LMICs), due to lack of specialized screening facilities and lack of trained health professionals to diagnose and manage these diseases [8, 9]. Consequently in Africa, many children die undiagnosed, or if diagnosed they often do not receive qualified and sufficient treatment at the hospital. Some may subsequently die because the family cannot afford long-term medication. In addition, tertiary facilities and trained personnel for paediatric diabetes and other endocrine diseases had been virtually nonexistent. Therefore, there is need to put in place programs addressing the two key barriers to care for children with endocrine disorders in Africa: capacity building and access to treatment.

Kenya is one of the fastest growing nations in the world, and children under the age of 18 years represent 53% of the population [10]. The annual growth rate of Kenya's child population is 2.2% and every child born in Kenya has a right to the highest attainable standards of healthcare encompassing five key parameters of availability, accessibility, affordability, acceptability, and quality [11]. Despite this, child mortality rates in Kenya remain unacceptably high. Estimates indicate that there are 106,000 child deaths every year with an under five-mortality rank of 33 [12]. Part of this is due to lack of specialized paediatric services and lack of trained health professionals including paediatric endocrinologists. Until the year 2007, Kenya had only one (1) paediatric endocrinologist. Today there are eight (8) board certified paediatric endocrinologists and of these, seven (7) are graduates of the Paediatric Endocrinology Training Centre for Africa (PETCA). PETCA was launched in 2007 in Nairobi, Kenya, with a mission to provide and maintain high quality training, research, and clinical practice in the endocrinology subspecialty to paediatricians from all over sub-Saharan Africa. Being one of a kind in Africa the PETCA initiative has been able to achieve great success and have a wide reach despite the limited resources available. The programme is run at the Gertrude's Children's Hospital, Kenya, in partnership with the University of Nairobi, Kenyatta National Hospital, and the Aga Khan University Hospital, Nairobi.

Gertrude's Children's Hospital is the longest serving paediatric health institution in East and Central Africa. Established in 1947, the hospital is dedicated to providing care and treatment exclusively to children. Gertrude's has a main hospital campus located in Muthaiga and various satellite clinics across the country. Over the years, the hospital has witnessed a dramatic expansion and now provides a total of over 300,000 visits annually. Many of these hospital visits are at the various local satellite clinics and are focused mainly on the provision of primary care to children. With the increase in outpatient attendees and inpatient admissions and vast expansion of its network, Gertrude's Children's Hospital was well placed to understand and set out to address the urgent need for paediatric specialists in Africa. This led to the launch of PETCA, a fellowship program which specifically aims to (1) equip paediatricians in Africa with the knowledge and skills to diagnose and manage childhood diabetes and other endocrine disorders and (2) facilitate the establishment of comprehensive centers of excellence, specializing in paediatric diabetes and endocrinology across sub-Saharan Africa. Here we report on the successes of the PETCA program, which will ultimately translate to the improvement of the quality and access to specialized healthcare to all children in sub-Saharan Africa.

## 2. Methods

### 2.1. PETCA Initiative

The Paediatric Endocrinology Training Center for Africa (PETCA) is a joint initiative of the Gertrude's Children's Hospital, Aga Khan University Hospital, Kenyatta National Hospital, University of Nairobi Department of Child Health, International Society for Paediatric and Adolescent Diabetes (ISPAD), and the European Society of Paediatric Endocrinology (ESPE). The program activities started running in January 2008 supported by funding from the World Diabetes Foundation.

### 2.2. Training Centre

The main training site is the Gertrude's Children's Hospital in Nairobi, Kenya, an institution dedicated to the provision of care and treatment exclusively to children. The hospital operates a busy inpatient unit and is building a model of outreach into the wider community.

### 2.3. Program Fellows

Fellows are candidates who already have National Medical Board recognition of specialist training in paediatrics in their home countries and for the PETCA program; they are recruited through a competitive process. Announcement and invitation for applications are made through regional medical associations, academic institutions, and tertiary hospitals in Sub-Saharan Africa countries. Applications are processed by the local faculty sitting in Kenya which ensures that equal opportunities are given to all regardless of age, gender, and race.

### 2.4. Fellowship Program Design

The fellowship is undertaken over an 18-month period, which begins with six (6) months of clinical and theoretical training in Kenya, nine (9) months of project research at the fellow's home country, and three (3) months consolidation in Kenya. Training comprises didactic sessions covering the length and breadth of paediatric endocrinology and diabetes, lectures and journal clubs, clinical teaching in the hospital clinics, and bedside as well as specific courses in research, leadership, and management.

### 2.5. Program Faculty

Tutorship was initially offered exclusively by ESPE and ISPAD international tutors. However, with the goal of self-sustainability the tutorship was later taken up increasingly by the local faculty, many of them alumni of the center. On completion, certification of the PETCA fellowship is by ESPE. The certified paediatricians are then expected to set up Centers of Excellence in their home countries and/or strengthen existing centers.

### 2.6. Role of Funding Source

The funding source had no role in the study design; in collection, analysis, and interpretation of data; in the writing of the report; and in the decision to submit the paper for publication.

## 3. Results

Since the launch of PETCA in 2008, there have been two successful phases, phase one which commenced in January 2008 through to October 2012 and phase two which started in January 2012 and was on until April 2015. Fifty-four (54) fellows have been certified: 34 (phase one) and 20 (phase two). The training program aims to include fellows from all regions in Africa: East, West, North, and South Africa. The fellows were from 12 African countries: Botswana, Congo, Ethiopia, Ghana, Kenya, Mauritius, Nigeria, Rwanda, Sudan, Tanzania, Uganda, and Zambia (Table 1).

During the PETCA fellowship, the fellows screened and treated paediatric patients who presented with a wide range of endocrine disorders including diabetes mellitus, obesity, stunted growth and development, pituitary disorders, failure to thrive, and thyroid disorders. These were patient referrals from paediatricians and general practitioners at the Gertrude's Children's Hospital outpatient department and from outreach activities including school screening drives. Initial patient enrollment was low due to lack of awareness and publicity in Kenya on the establishment of clinics in the three participating hospitals. Once this was done through various scientific meetings and trainings, the numbers have increased tremendously. To date, a total of 1,399 patients have been screened and treated since the launch of PETCA in 2008 (Table 2).

On completion of the PETCA training, all fellows are expected to return to their home countries and establish or strengthen existing endocrinology clinics. Eight (8) clinics have been strengthened. These are located in Kenya, Tanzania, and Sudan and 23 clinics have been established across eight (8) countries across Africa, all dedicated to the paediatric endocrinology subspecialty (Table 3).

Additional achievements of PETCA include (1) the initiation of the “Growth in Practice” (GIP) and “Diabetes in Practice” (DIP) training courses which are conducted with an aim of sensitizing paediatricians, nurses, and nutritionists on taking and interpreting growth measurements correctly. The healthcare workers are also trained on how to link these indices to paediatric endocrinology disorders and diabetes. More than one hundred healthcare workers have undertaken these courses to allow for the provision of high quality care closer to where patients live. (2) The fellows are also active in marking the World Diabetes Day in collaboration with the Diabetes Kenya Association. (3) Media coverage on PETCA has resulted in increased referrals and some patients have been adopted under the needy cases for support (http://www.gertrudeshospitalfoundation.org/). (4) Through partnership with the “Changing Diabetes in Children” initiative, PETCA alumni in Kenya, Uganda, and Tanzania have rolled out a training program known as the Changing Diabetes in Children (CDiC) program for health workers in lower level health facilities located in Nakuru, Nyeri, Mombasa, Malindi, Embu, Kisii, Kakamega, Mombasa, and Nairobi's Mama Lucy Hospital in Kenya. (5) There is increased access to essential endocrine drugs at these nine CDiC centers, where they provide free insulin, glucometers, glucose strips, and hemoglobin A1c (HbA1c). Furthermore, hydrocortisone tablets are now freely available and thyroxine is part of the government hospital kit provided at the Kenyatta National Hospital. (6) As a result of the success of PETCA in Nairobi, a similar program, PETCA-West Africa, was launched in Lagos, Nigeria, in 2013 to expand training opportunities for paediatricians in West Africa. (7) The program has benefitted from free genetic analysis that is done in Exeter, United Kingdom, for all countries if the patient (child or adult) with diabetes has had an onset before the age of six months. (8) There are currently ongoing discussions on having the program being officially recognized by the University of Nairobi in Kenya. This will add to the PETCA fellowship international credibility and continuation of the program is planned even after expiration of the World Diabetes Foundation grant with tutors from ESPE and ISPAD.

## 4. Discussion

This is the first report on a successful paediatric endocrinology fellowship program in Africa. PETCA is a fellowship program that was launched to specifically equip paediatricians with the knowledge and skills to diagnose and manage childhood endocrinology disorders and diabetes. Africa has inadequate paediatric subspecialist manpower and this concern is not unique to the region [13–15]. In fact, the American Academy of Paediatrics in 2013 reiterated that there is a shortage of paediatric medical subspecialists in USA and the current pool of paediatricians is insufficient to serve the growing need for family-centered care [16]. These sentiments are echoed in Asia and Europe [17, 18]. PETCA is therefore well placed to contribute to the growth of the paediatric workforce pool in Africa and on the global front at large.

A recent report by the Global Paediatric Education Consortium (GPEC) indicates that the presence of a paediatric workforce in a nation can significantly impact the quality of life for children [19]. The PETCA initiative is a concerted effort to address the workforce disparity across Africa. Fifty-four fellows have graduated from the program since its launch in 2008, from twelve African countries: Botswana, Congo, Ethiopia, Ghana, Kenya, Mauritius, Nigeria, Rwanda, Sudan, Tanzania, Uganda, and Zambia. The fellows are committed to ensuring equitable, affordable, and accessible provision of paediatric healthcare services across all regions as witnessed in the number of clinics that have been set up or strengthened following completion of both phases one and two of the program. Eight clinics have been strengthened, located in Kenya, Tanzania, and Sudan, while 23 clinics have been established in eight countries across Africa, all dedicated to the paediatric endocrinology subspecialty.

During the fellowship, PETCA fellows had the opportunity to diagnose, treat, and create awareness on diabetes and endocrinology disorders. The findings of this report demonstrate that diabetes and obesity were the leading disorders in this setting accounting for 72% of all cases. Thyroid disorders were the least common among two percent of patient pool. The noncommunicable disease (NCD) epidemic in Africa is fast growing but silent and ignored [20, 21]. There is great paucity in the data, especially on the burden and prevalence rates of diseases, because of misdiagnosis, underdiagnosis, and poor health systems infrastructure in most African countries [22]. Nonetheless, diabetes and obesity are recognized as leading causes of mortality and morbidity in children in Africa [23–25]. Therefore, concerted efforts by PETCA to build capacity within the region will translate in a significant reduction in endocrine- and diabetes-linked morbidity and mortality rates which will go a long way in addressing the wide disparity in the quality of life for children around the world [26, 27]. In addition, this will encourage an increase in investments channeled towards paediatric endocrinology.

Child health needs are unique and complicated as children are constantly developing. Therefore, paediatricians are the best physician specialists to address their health needs. Paediatric endocrinologists are subspecialists with additional focused training in the specialized area of endocrinology. PETCA advocates and caters for the need for paediatric endocrinology subspecialists and these efforts have had a transformational impact on the lives of children in SSA. Practical instruction during the fellowship has allowed actual detection, treatment, and care of patients with complex endocrine conditions and diabetes within the training institutions. The expertise required to diagnose and manage the complex child endocrine disorders is not part of routine paediatric training. Hence, building capacity of endocrinology specialists across Africa provides a crucial network base for referrals and consult services for clinicians in tertiary facilities, managing complex disorders. Prompt identification of the disorders and treatment has been shown to have a significant impact as it prevents the detrimental lifelong physical and neurodevelopmental effects for the child [1]. It is therefore very important that the care provided should be by a specialist experienced in paediatric endocrinology management, so as to address the complexity of the evaluation adequately.

PETCA is a sustainable initiative, as the alumni are now taking part as trainers so as to ensure the capacity building efforts, specifically training new fellows, and the successes of the PETCA program will translate to the improvement of the quality and access to specialized healthcare to all children in Sub-Saharan Africa. Overall, PETCA efforts are in line with internationally recognized recommendations and guidelines including the British Society for Paediatric Endocrinology and Diabetes, Australasian Paediatric Endocrine Group, and the Canadian Paediatric Endocrine Group [28–30]. All these are concerned with the management and research that will foster a better understanding of disorders of the endocrine system in children. They each have training opportunities, similar to PETCA, which are in place to ensure that paediatricians are well trained on child endocrinology and diabetes and ensure equity in capacity globally.

## 5. Conclusion

The successes of the PETCA program demonstrate the impact a capacity building and knowledge transfer model can have on a population. PETCA serves as an innovative model to encourage equity in the availability of qualified health care personnel globally. The model provides more training opportunities than would have been available in a developed country. Training on the continent also means all qualified personnel are retained on the continent.

## Figures and Tables

**Table 1:** Number of fellows trained from the PETCA initiative during phases one and two (January 2008–April 2015).

Country	Number of fellows		
	Phase one (complete)	Phase two (complete)	Total
	January 2008–October 2012	January 2012–April 2015	
(1) Botswana	2	—	**2**
(2) Congo	—	1	**1**
(3) Ethiopia	1	—	**1**
(4) Ghana	1	—	**1**
(5) Kenya	6	3	**9**
(6) Mauritius	—	1	**1**
(7) Nigeria	11	4	**15**
(8) Rwanda	—	1	**1**
(9) Sudan	5	7	**12**
(10) Tanzania	6	—	**6**
(11) Uganda	2	2	**4**
(12) Zambia	—	1	**1**
Total	**34**	**20**	**54**

**Table 2:** Number of patients screened and treated during the PETCA fellowship from 2008 to 2015.

Year	Diabetes	Obesity	Stunted development	Pituitary	Failure to thrive	Thyroid disorders	Total
2008	77	10	10	8	2	1	**108**
2009	154	36	26	15	15	0	**246**
2010	8	9	5	1	11	3	**37**
2011	141	46	22	11	19	5	**244**
2012	98	45	13	16	32	7	**211**
2013	69	15	19	21	17	4	**145**
2014	112	26	19	14	16	9	**196**
2015	132	33	13	18	15	1	**212**
Total	**791**	**220**	**127**	**104**	**127**	**30**	**1399**

**Table 3:** Clinics strengthened and established by graduates of the PETCA initiative.

Strengthened clinics	
Country	Institution

Kenya	Kenyatta National Hospital

Tanzania	Kilimanjaro Christian Medical Centre
	Muhimbili National Hospital

Sudan	Abu Se'id Paediatric Hospital
	University of Gezira Teaching Hospital
	Ministry of Health
	Fedail Hospital, Khartoum Gaafar Ibnauf Specialised Children's Hospital

Established clinics	

Country	Institution

Kenya	Gertrude's Children's Hospital
	Aga Khan University Hospital
	Thika Level 5 District Hospital
	Machakos Level 5 District Hospital

Nigeria	Federal Medical Centre, Gombe
	Benin Teaching Hospital
	University Teaching Hospital of Ilorin
	Obafemi Awolowo University and its Teaching Hospital
	Federal Medical Centre in Asaba
	Olabisi Onabanjo University Teaching Hospital
	Federal Teaching Hospital Abakaliki, Ebonyi State
	University of Calabar Teaching Hospital

Botswana	Baylor Children's Clinical Centre of Excellence
	Princess Marina Hospital

Ghana	Komfo Ankoye Teaching Hospital

Ethiopia	Black Lion Hospital

Tanzania	St. Francis Referral Hospital

Sudan	Abu Se'id Paediatric Hospital

Zanzibar	Mnazi mmoja Referral Hospital

Uganda	Makerere University
	Mulago Hospital
	Gulu Regional Hospital
	Kagando Hospital
	Rubaga Hospital
